# Supplementary material for: What is the impact of flexicurity on the chances of entry into employment for people with low education and activity limitations due to health problems? A comparison of 21 European countries using Qualitative Comparative Analysis (QCA)
Source: BMC Public Health. 2016 Aug 19;16:842. doi: 10.1186/s12889-016-3482-2 (PMC4992207; doi:10.1186/s12889-016-3482-2)
Supplement: Additional file 1: — Calibration of conditions. (DOCX 27 kb) [file 12889_2016_3482_MOESM1_ESM.docx]

## Additional file 1 - Calibration of conditions

**Outcomes**

For RTW, percentages range from as low as 4 % (Ireland) to 34 % (Norway). To put these figures in perspective, the target for work-related rehabilitation in Sweden is 40 % [[1](#_ENREF_1)] and 35 % was achieved in 2005-08. 35 % is also the (pre-crisis) average employment rate of people with severe disability in the EU (EC, 2004). Thus, 35 % is set as the higher threshold (this is not achieved by any country), and 9 % (n 5) as the lower threshold. The 0.5 anchor is set at a gap in the data, dividing Hungary (14 %) from Portugal (12 %).

For the rate ratio, scores range between 0.35-0.99. Those above 0.8 (n 3) are coded as fully in the set of equal countries, while those scoring below 0.5 (n 5) are coded as fully out. Countries scoring above 0.66 were coded as more in than out (n 11), dividing the Czech Republic (0.67) and Estonia (0.65)^[[1]](#footnote-1)^.

**Background conditions**

*Employment*

The employment rate is calculated as the average during 2005-2010 in the population 20-64 years. Average employment is likely to strongly affect the probability of return to work and varies between 80 % (Norway) and 62 % (Hungary). It is difficult to say at what point full employment is reached, as this depends on labour supply as well as demand. However, based on the Europe 2020 target, we have defined those countries with 75 % or above as having high employment (n 4). Those with employment rates below 63 % are considered as fully out (n 3), based on the lowest target set for an individual country (http://ec.europa.eu/europe2020/pdf/targets_en.pdf). Different thresholds were tested and eventually the 0.5 anchor is set in between 71, dividing Ireland (more out than in) and the Czech Republic (more in than out).

*Average unemployment*

Average unemployment during 2005-2010 in the population 20-64 years was considered as an alternative background indicator. There seems to be no general standard regarding what may be considered high or low unemployment. While an unemployment near zero is not desirable, as this would imply a completely rigid labour market, it does appear that the NAIRU (non-accelerating inflation rate of unemployment) may be compatible with both low and high structural (long-term) unemployment rates [[2](#_ENREF_2)]. Average unemployment varies from 2.8 % in Norway, to 12 % in Slovakia with an overall average of 7 %. Countries scoring 5 % or less are considered as fully in the set of low unemployment countries. Five countries fulfill this criterion: Norway, Austria, Denmark, Luxembourg and the Netherlands. Countries scoring 9 % or above are coded as fully out (N=4). The point of maximum ambiguity is set at 7 % where a natural gap in the data occurs between Italy (6.95 %) and Ireland (7.3).

**Policy conditions**

*EPL - Employment protection legislation*

For Employment protection legislation (EPL), indicators used are those collected and coded by the OECD^^[[2]](#footnote-2)^^. The index consists of three dimensions and 21 indicators. The dimensions are:

- Individual dismissals of workers with regular contracts (EPR)
- Temporary employment (EPT)
- Additional regulations for collective dismissals (EPC)

Due to the nature of our study group i.e. people not currently in employment with activity limitations, we focus on the first two dimensions as more relevant. Following Emmenegger [[3](#_ENREF_3)] we have slightly altered the coding of the dimensions. For temporary contracts, the minimum rather than the mean of the two included indicators is used. As the two types of employment - temporary work agencies and fixed-term contracts - are functionally equivalent to the employer, the one less regulated should be the one preferred. Also, previous research has shown that one of three indicators used for regular contracts, ‘difficulty of dismissal’, is a better predictor of job-loss and incidence of unemployment [[4](#_ENREF_4)], thus it has been given greater weight (from ^1^/_3_ to ^1^/_2_) than the other two (from ^1^/_3_ to ^1^/_4_). In contrast to Emmenegger, and again due to our particular study question, we have retained an equal weighting between temporary and regular contracts, as the former may be of special importance to outsiders. In subsequent analyses, EPL (average of EPT and EPR) and EPT are tested as two alternative conditions.

We have calibrated the set of low-regulation countries as follows. Only one country scores 1 or below (=fully in) on EPL: the UK (with a score of 0.74). The next lowest is Ireland at 1.24. Two score above 3, France and Luxembourg. Although the score theoretically ranges from 0-6, no country scores above 3.8 (Luxembourg on EPT).The fully out threshold, 0, is therefore set at 3.5. The point of maximum ambiguity is set at 2. Thus, Finland is considered more in than out, and Austria more out than in, which is in agreement with Emenegger’s coding. For EPT, the cases are much more dispersed, with six countries scoring below 1. In general, countries are much less regulated for this indicator. For EPT, the same threshold (3.5) is used for fully out, while the threshold for fully in is 0.7. The point of maximum ambiguity is set at 1.4, between Finland and Estonia (1.33) and Slovenia and Belgium (1.5).

*Active labour market policy*

For ALMP, expenditure varies from close to nil to at most 0.3 % of GDP per percentage unemployed. Relating this figure to the average unemployment 2005-10 in included countries (7 %), ALMP expenditure ranges from 0.04 % of GDP (Estonia) to 2.1 % (Denmark). We considered those countries that spend 0.02 % or less of GDP (n 5) as being fully out and those spending 0.2 % or above (n 2) as being fully in. The crossover point is set at 0.07 %, between France (0.081 %) and Poland (0.066 %).

For PES too we find that expenditure is highly skewed. Countries spend from almost nothing to 0.11% (The Netherlands) of GDP per percentage unemployed. Half of all countries actually spend less than 0.02 %. With an average unemployment of 7 %, this corresponds to a range between 0.01 % (Greece) and 0.76 % of GDP. PES is clearly a cheaper service than active measures, thus to be fully in requires no more than 0.06 % of GDP (n=2). This level was reached only by Sweden and Denmark out of six west European countries in 1985-2005 [[5](#_ENREF_5)], while the lower threshold is set at 0.007 % (n 3). The 0.5 point threshold is set in a natural gap in the data, between France (0.026 %) and Slovenia (0.019 %).

ALMP and PES expenditure was also added into the summary measure ALMPtot. The chosen thresholds for this indicator was 0.3 % for being fully in, 0.02 % for being fully out, and 0.07 % for the 0.5 threshold.

*Social security*

Passive labour market policy, measured by expenditure on unemployment benefit as a percentage of GDP per percentage unemployed, is part of the security dimension of flexicurity, and the generosity of unemployment benefits should ideally be coupled with active measures. To relate to ALMP, we use the same thresholds for being fully in (n 2) and fully out (n 2). The 0.5 anchor is set at a gap in the data, between Italy (0.11 %) and Luxembourg (0.08 %).

Theoretically, sickness benefit is part of flexicurity as a temporary alternative to labour market participation. This indicator is standardized to the proportion with activity limitations among those aged 45-64. Two countries, Norway (0.24 %) and Sweden (0.13 %) score higher than the rest. The fully in threshold is set at 0.1 % of GDP (n=2) and the fully out threshold is set between Hungary (0.033) and Estonia (0.039). The point of maximum ambiguity is set in a gap in the data, between Austria (0.056) and Slovenia (0.059).

*Social services expenditure*

Social services expenditure as a percentage of GDP, standardized to the percentage under 5 and over 64 years of age, varies substantially, from practically zero to 0.50 per percentage point in the population. Usually much more is spent on child care than on the elderly, with exceptions being Slovakia (where less is spent on child care) and Ireland and the Netherlands (where spending on child care is only slightly higher). This is not surprising given that only a minority of those 65 and over require care. Although there is some discrepancy between the two indicators, the high and low performers tend to be the same. The main difference is that many more countries spend very little on the elderly.

The two indicators were tested both singly and combined into a summary score. Expenditure on services directed at families varies from nil (Luxembourg) to 0.27 % (Sweden). The Nordic countries (n 4) score above 0.2 %, which is set as the fully in threshold, and 0.05 % is set as fully out (n 5). There is no obvious gap in the data. 0.1 % is set as the 0.5 threshold, dividing Portugal (0.092 %) and Greece (0.103 %).

Much less is spent on elder care (per percentage 65+), as mentioned. In fact, seven countries spend less than 0.01 % which is set as the lower threshold. Three countries spend over 0.1 % which is the higher threshold. Sweden has the highest expenditure (0.14 %). In between these points the countries are quite evenly spread. The 0.5 threshold is set between Austria (0.026 %) and Hungary (0.030 %)

The distribution of the additive social services indicator is highly skewed towards the left. We have chosen to code the Nordic countries as being fully in the set of high spenders, setting the threshold at 0.3 % (n 4). Those scoring below 0.1 % are set as fully out (n 6). In between the extremes there is no obvious threshold; however we have chosen to distinguish between Italy (0.13) and the Netherlands (0.14). The latter spends on both child and elder care [[6](#_ENREF_6)], while Italy spends more generously on child care and almost nothing on elder care. The Netherlands is also often clustered with the Nordic countries in terms of generosity and universalism.

Life-long learning

Unlike the other policy indicators, life-long learning is more an outcome of policy and does not distinguish between efforts made by employers and those made by Government. In some respect, it may be seen as a labour market condition in the same way as the employment rate. Variation between countries is large, from Romania with less than 2 %, to Denmark at 30 %. The EU benchmark is that at least 15 % of the population in the age group 25-64 should participate in life-long learning, particularly those who are low-skilled (http://ec.europa.eu/education/dashboard/index_en.htm). Countries above 20 % (n=3) have been coded as fully in and those below 5 % (n=4) as fully out. The 0.5 anchor is set between Luxemburg (8.5 %) and Estonia (9.8 %).

**References**

1. Johansson P, Nilsson M. Should sickness insurance and health care be administrated by the same jurisdiction? An empirical analysis. Uppsala: IFAU2012.

2. Srinivasan N, Mitra P. Hysteresis in unemployment: Fact or fiction? Economics Letters. 2012;115(3):419-22. doi:10.1016/j.econlet.2011.12.070.

3. Emmenegger P. Job security regulations in Western democracies: A fuzzy set analysis. European Journal of Political Research. 2011;50:336-64. doi:10.1111/j.1475-6765.2010.01933.x.

4. Bertola G, Boeri T, Cazes S. Employment protection and labour market adjustment in OECD countries: Evolving institutions and variable enforcement. . Paris: International Labour Organisation1999.

5. Bonoli G, Emmenegger P. State-Society Relationships, Social Trust and the Development of Labour Market Policies in Italy and Sweden. West European Politics. 2010;33(4):830-50. doi:10.1080/01402381003794647.

6. van Hooren F, Becker U. One Welfare State, Two Care Regimes: Understanding Developments in Child and Elderly Care Policies in the Netherlands. Social Policy & Administration. 2012;46(1):83-107. doi:10.1111/j.1467-9515.2011.00808.x.

1. Initially, 0.7 was set as the threshold. However, this excludes the Czech Republic, which was consistently included in the solution, regardless of what conditions were tested. Our decision to resolve the contradiction was to adjust the threshold slightly. [↑](#footnote-ref-1)
2. For non-OECD countries, some data is available from the fRDB-IMF Labour Institutions Database. However, the only indicators included are the advance notice period and the severance pay after certain employment durations, item 2 out of the 9 items that make up the EPR. [↑](#footnote-ref-2)
