# Supplementary material for: What is the impact of flexicurity on the chances of entry into employment for people with low education and activity limitations due to health problems? A comparison of 21 European countries using Qualitative Comparative Analysis (QCA)
Source: BMC Public Health. 2016 Aug 19;16:842. doi: 10.1186/s12889-016-3482-2 (PMC4992207; doi:10.1186/s12889-016-3482-2)
Supplement: Additional file 3: — Most complex and most parsimonious solution. (DOCX 14 kb) [file 12889_2016_3482_MOESM3_ESM.docx]

## Additional file 2 - Most complex and most parsimonious solution

Outcome 1 RTW

COMPLEX SOLUTION

Combination 1

empave_ny*almp_cal*plmp_sick*old_cal

Denmark (0.97,0.92), Norway (0.91,0.95), the Netherlands (0.78,0.57), Finland (0.69,0.8), Sweden (0.69,0.91)

consistency 0.80 coverage 0.57

Combination 2

empave_ny*ept_cal*~almp_cal*~plmp_sick

United Kingdom (0.8,0.75), Estonia (0.57,0.69), Czech Republic (0.56,0.66)

consistency 0.74 coverage 0.35

Combination 3

~empave_ny*~ept_cal*~almp_cal*~plmp_sick*old_cal

Hungary (0.51,0.53)

consistency 0.81 coverage 0.12

PARSIMONIOUS SOLUTION

Combination 1

empave*old_cal

Sweden (0.99,0.91), Norway (0.98,0.95), Denmark (0.97,0.92), Finland (0.8,0.8), Netherlands (0.78,0.57), United Kingdom (0.55,0.75)

consistency 0.80 coverage 0.69

Combination 2

empave*ept_cal

Sweden (0.96,0.91), Netherlands (0.94,0.57), United Kingdom (0.94,0.75), Denmark (0.57,0.92), Estonia (0.57,0.69), Finland (0.57,0.8), Czech Republic (0.56,0.66)

consistency 0.71 coverage 0.71

Combination 3

~ept_cal*~plmp_sick*old_cal

Hungary (0.51,0.53)

consistency 0.82 coverage 0.14

**Outcome 2 RR**

COMPLEX SOLUTION

Combination 1

uneave_ny*almp_cal*plmp_sick*socexp_cal

Denmark (0.97,1), Norway (0.91,1), Sweden (0.69,0.94), Finland (0.54,0.75), Austria (0.52,0.78), the Netherlands (0.51,0.9)

consistency 0.98 coverage 0.45

Combination 2

uneave_ny*epl_cal*~almp_cal*~plmp_sick*~socexp_cal

United Kingdom (0.77,1), Czech Republic (0.63,0.58)

consistency 0.94 coverage 0.24

Combination 3

uneave_ny*~epl_cal*~almp_cal*plmp_sick*~socexp_cal

Luxembourg (0.56,0.74), Slovenia (0.53,0.91), Italy (0.52,0.91)

consistency 0.92 coverage 0.22

PARSIMONIOUS SOLUTION

uneave_ny

The Netherlands (1,0.9), Norway (1,1), Denmark (0.98,1), Luxembourg (0.98,0.74), Austria (0.97,0.78), United Kingdom (0.94,1), Slovenia (0.86,0.91), Czech Republic (0.75,0.58), Sweden (0.73,0.94), Finland (0.54,0.75), Italy (0.52,0.91)

consistency 0.87 coverage 0.82
